# Leader–Follower Congruence in Work Engagement and Leader–Member Exchange: The Moderating Role of Conscientiousness of Followers

**DOI:** 10.3389/fpsyg.2021.666765

**Published:** 2021-07-27

**Authors:** Yanhua Ye, Ziwen Wang, Xiaowei Lu

**Affiliations:** ^1^School of Business Administration, Zhejiang Gongshang University, Hangzhou, China; ^2^School of Nursing, Jinan University, Guangzhou, China

**Keywords:** work engagement, LMX, conscientiousness, person-environment fit, polynomial regression

## Abstract

Extant research has investigated the relationship between work engagement and various outcomes, such as job performance and organizational commitment, neglecting the effect of work engagement on social relationships at work. Drawing upon person-environment fit theory and LMX theory, the present study aims to examine the effect of (in)congruence between leader and follower work engagement on leader–member exchange (LMX) and the moderating effect of conscientiousness. About 273 employees and 72 leaders participated in this study and completed the measurements of work engagement, conscientiousness, and LMX at two time points. Using cross-level polynomial regressions, we found that, compared with incongruent work engagement, employees perceived high levels of LMX quality when their work engagement was aligned with that of their leaders. Regarding the congruence, the employees reported higher levels of LMX when congruence in work engagement was at higher rather than lower levels. Regarding the incongruence, when the employees engaged less in their work tasks than their leaders, they were more likely to experience lower LMX. Moreover, the negative relationship between incongruence in leader and follower work engagement and LMX was mitigated when followers were more conscientious. All our hypotheses were supported. Both theoretical and practical implications for work engagement as well as future directions are discussed.

## Introduction

In the last two decades, the number of studies on work engagement has increased rapidly (Bakker and Albrecht, 2018). Work engagement is defined as “a positive, fulfilling, work-related state of mind that is characterized by vigor, dedication, and absorption” (Schaufeli et al., 2002, p. 74). People who engage in their work show high levels of energy and involvement in work-related activities, viewing their work as more interesting and meaningful (Harju et al., 2016), and holding more positive effects in terms of their work roles (Bakker and Demerouti, 2008). In addition, although it is a work-related state of mind, the literature on work engagement has suggested that this is a relatively stable variable and can be used to predict outcomes across time (Macey and Schneider, 2008). Research on work engagement has suggested that work engagement relates to various positive outcomes, such as higher job performance (Breevaart et al., 2015) and higher organizational commitment (Demerouti et al., 2001).

Although various outcomes have been examined in extant studies, there is still a critical research gap. Previous studies mainly focused on how work engagement influences work-related performance and attitudes, neglecting its effects on social relationships with others (e.g., leaders) in the workplace. Actually, work engagement, as an important motivational resource (Kim et al., 2018), may influence the work attitudes of others, which, as a consequence, may establish and maintain connections with others (Bakker and Xanthopoulou, 2009). Compared with social relationships with coworkers, social exchange relationship of employees with their supervisor (LMX) may have far-reaching influences on employees due to the greater authority and power of leaders (Agle et al., 2006). Thus, it is important to explore the relationship between work engagement and LMX. In addition, practically, employees do not live in a social vacuum. Engaged employees may not always perceive high levels of LMX. Leaders are crucial sources of support and play critical roles in influencing social relationships (Gutermann et al., 2017). For example, if the work engagement of a follower is high while the work engagement of a leader is low, employees tend to evaluate their leaders as unattractive and dissimilar from them, ultimately diminishing their perceived LMX. Therefore, exploring the relationship between work engagement (in)congruence and LMX is theoretically and practically important because doing so extends the effects of work engagement beyond work-related performance and attitudes and enriches our understanding of work engagement.

In the current study, we draw from person-environment fit theory and LMX theory to examine whether congruence (or incongruence) in leader and follower work engagement may help (or inhibit) followers develop high-quality LMX. We also aim to test when the relationship between incongruence in leader and follower work engagement and LMX would be stronger and weaker. Previous researchers suggested that, when exploring the effects of a person-environment fit, individual personalities should also be considered (Harms et al., 2006). Among various personalities, conscientiousness, defined as personal characteristics, such as persistence, planfulness, carefulness, and responsibility (Barrick and Mount, 1991; Humberg et al., 2019), has been uniformly regarded as a valuable personal trait that contributes to various benefits at work (Judge and Ilies, 2002; Parks-Leduc et al., 2015). Indeed, conscientiousness has been identified as one of the worthiest personal traits that should be studied in most organizational settings (Brown et al., 2011). In addition, according to its definition, conscientious individuals are more likely to persist and exert more effort and time to fulfill their role demands and help the development of LMX (Lapierre and Hackett, 2007). As such, conscientiousness may mitigate the negative relationship between incongruence in follower and leader work engagement and LMX.

Taken together, hypothesizing and testing these relationships would contribute to the theory and research on work engagement in three main ways. First, this study fills a critical research gap in previous studies in terms of the relationship between work engagement and LMX, which extends the theoretical framework of work engagement. Previous studies of work engagement mainly focus on outcomes beyond LMX (Breevaart et al., 2015). Despite the significance of examining the effects of work engagement on other outcomes, exploring the relationship between work engagement and LMX will help us have a more comprehensive understanding in terms of work engagement as LMX is a greatly important indicator of leader–member relationship and relates to various outcomes, such as performance (Park et al., 2015; Martin et al., 2016).

Second, we contribute to research on the effects of work engagement by showing that not only simply the work engagement of employees but also the combination of leader and follower work engagement plays important roles in shaping LMX. Scholars have called for research to investigate how (in)congruence in leader and follower personal traits or work attitudes influences work-related outcomes (Zhang et al., 2012). A majority of studies have investigated the effects associated with (in)congruence in leader–follower personal traits (such as proactive personality, Xu M. et al., 2019). There are only a few studies that focused on the effects of (in)congruence in leader and follower work-related attitudes (Claudia et al., 2009). Exploring the effects of (in)congruence in leader and follower work engagement on LMX provides us insights to understand the relationship between work engagement and LMX from a person-environment-fit perspective.

**Figure 1:**
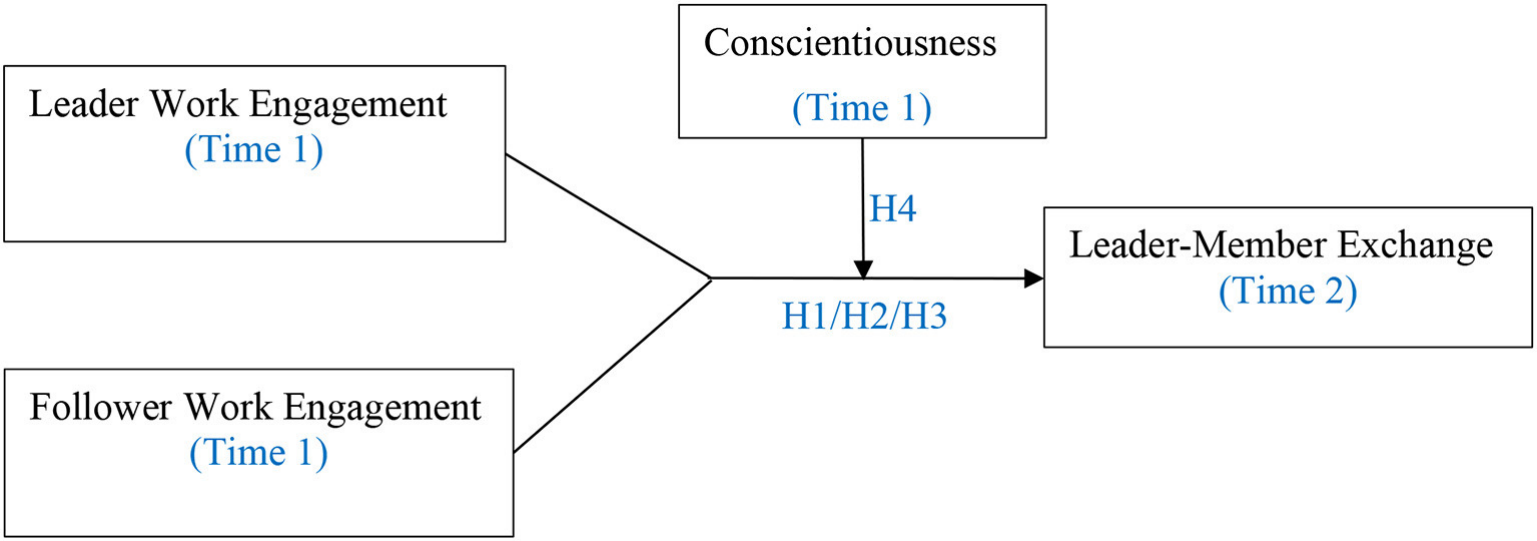
A hypothesized model.

Third, we identify an important personality trait (i.e., conscientiousness) as a key boundary condition in buffering against the detrimental effect of incongruence in leader and follower work engagement on LMX. Previous studies greatly demonstrated the detrimental effects associated with the incongruence effect and rarely tested potential boundary conditions (e.g., Zhang et al., 2012; Riggs and Porter, 2016; Yang et al., 2017). Actually, scholars should pay attention to how to mitigate the negative effects of the incongruence effect (Follmer et al., 2018). Our study wants to make a further step to identify a dispositional factor in alleviating the incongruence effect of leader and follower work engagement on LMX. Figure 1 depicts the hypothesized model.

## Theoretical Basis and Hypothesis

### Leader–Follower (In)Congruence in Work Engagement and LMX

In this study, we draw upon person-environment fit theory and LMX theory to examine the effect of leader and follower (in)congruence in work engagement on LMX. According to the person-environment fit theory (Kristof-Brown et al., 2005), the person-supervisor fit will result in considerable positive outcomes (Schaubroeck and Lam, 2002; Kristof-Brown et al., 2005). A fit between leaders and followers regarding some characteristics (e.g., personality traits and work engagement) will provide followers with increased supervisor support due to the similarity (David et al., 2010). At the same time, the mutual coordinated interactions and trust will be established via a person-supervisor fit (Thompson et al., 2006). All these factors are beneficial for the development of LMX. Indeed, research has suggested that LMX is theoretically relevant to the dyadic exchange relationship between a leader and a follower and is often viewed as a proximal outcome of a person-supervisor fit (Graen and Uhl-Bien, 1995).

According to LMX theory, LMX is a dyadic relationship between leaders and followers. It is developed over time through three stages: role taking, role making, and role routinization. Role taking is the initial interaction process whereby leaders send roles to followers and evaluate their reactions. Considering leaders are not familiar with followers in the early stage of the relationship, leaders may evaluate them via other characteristics, such as gender and work-related attitudes (Bauer and Green, 1996). Similarities in those characteristics between leaders and followers will increase mutual attraction and trust, resulting in high evaluations from leaders (Turban and Jones, 1988; Meglino et al., 1989). At the role making stage, leaders will further send roles to followers if they evaluate those followers as favorable and meeting their expectations. Simultaneously, followers are not to passively accept role assignments but to actively engage in their roles and assess the reactions of their leaders (Graen and Scandura, 1987). Finally, in the role routinization phase, the relationship between leaders and followers becomes formalized and affect laden. Solid relationships between leaders and followers are established at this stage. Through dynamic interactions in the three processes, LMX is developed (Graen and Scandura, 1987).

#### Comparing Congruence With Incongruence

According to the person-environment fit theory and the LMX theory, we propose that, compared with incongruence in leader and follower work engagement, congruence would lead to high levels of LMX. When follower work engagement and leader work engagement are aligned at the same levels, followers are more likely to experience a shared perspective on the meaningfulness and challenge of their work requirements (Harju et al., 2016). Moreover, drawing upon the person-supervisor fit theory, the similarity in terms of work engagement enables followers to obtain more support from their leaders, evaluating their leaders as more favorable and trust (Chen et al., 2016). In addition, a shared perception of work engagement will increase coordinated interactions between leaders and followers (Metiu and Rothbard, 2013; Costa et al., 2014) and drive followers to fulfill role requirements imposed by leaders. These shared perceptions and similarities promote the development of the role-taking and role-making processes of employees and make the relationship easier to enter into the role routinization process (Zhang et al., 2012; Chen et al., 2016). The congruence in leader and follower work engagement may further promote mutual trust and attraction (Thompson et al., 2006), thereby enhancing perceived relationship of employees with leaders. In contrast, compared with the beneficial effect of the congruent work engagement among leaders and followers on LMX, the incongruence in leader and follower work engagement is detrimental to perception of employees of LMX. Followers perceive a less-shared identical perspective of self-in-role, low supervisor support, and are less likely to cooperate with leaders, which inhibits the development of LMX.

##### Hypothesis 1

The more aligned the levels of work engagement of a follower and of his or her leader are (i.e., higher congruence), the better the LMX quality.

#### Comparing Two Subtypes of Congruence

Followers and leaders may be congruent at either higher or lower levels of work engagement. When an engaged employee teams up with an engaged leader, their common understanding of work significance may encourage them to establish a high-quality social relationship. In addition, a leader and a follower may have more positive interactions about their work tasks, which then increase mutual attraction and trust (Bakker and Xanthopoulou, 2009). Thus, a positive relationship between leaders and followers may be developed. In contrast, when followers and leaders are congruent at lower levels of work engagement, although both parties have a shared perception of work engagement, they may consider their work less important and valuable (Bakker et al., 2006). Thus, they may engage less energy and put less effort into their work-related tasks and activities, which may reduce meaningful interactions between leaders and followers (Bakker and Xanthopoulou, 2009; Gutermann et al., 2017). Based on LMX theory, less meaningful interactions will reduce the willingness to formalize role routinization (Matta et al., 2015). Then, solid relationships between leaders and followers will be restrained at this stage. In addition, less engaged followers will be less motivated to respond to role requirements of leaders (Deary et al., 2003; Harju et al., 2016; Wirtz et al., 2017), lowering evaluation of leaders of their followers. As such, according to the LMX theory, the development of role processes is inhibited (Dienesch and Liden, 1986; Zhang et al., 2012). Taken together, we propose:

##### Hypothesis 2

LMX quality is higher when a follower is aligned with a leader at higher than lower levels of work engagement.

#### Comparing Two Subtypes of Incongruence

LMX may also be affected differently by two scenarios of leader–follower incongruence in work engagement: when the work engagement of the leader is higher than that of the follower and vice versa. We expect that it is more detrimental to LMX quality when the leader has a higher level of work engagement than the follower. Specifically, when followers have lower levels of work engagement than their leaders, they may fail to supply resources and effort in both the role-taking and role-making phases of LMX development (Stewart and Johnson, 2009). Thus, according to LMX theory, engaged leaders become reluctant to send other role requirements to those disengaged followers and may evaluate the performance of their followers more negatively (Bauer and Green, 1996). Moreover, less engaged followers may feel stressed when working with leaders who have high levels of work engagement (Porter, 2001). Thus, they are prone to alienate and avoid more social interactions with their leaders (Westman and Chen, 2017). As a consequence, the development of the leader–follower relationship and interaction is greatly prohibited.

In contrast, when follower work engagement exceeds that of their leaders, despite less work-related interactions between leaders and followers, employees are still willing to complete role requirements assigned by the leaders and even engage more energy and efforts in extra work tasks, such as actively helping their leaders and coworkers. Thus, even though their leaders are less engaged, they may be more likely to assign some work tasks to those engaged workers because they believe these employees may work better (Xu A. et al., 2019). As such, these employees may obtain extra achievement and are more likely to receive positive evaluations from their leaders (Knight et al., 2017). According to LMX theory, positive evaluation from leaders may promote the development of role making and role routinization stages, which ultimately help establish a positive relationship between leaders and followers. As a consequence, the negative interaction and evaluation between employees and leaders may be mitigated. Therefore, we propose:

##### Hypothesis 3

LMX quality is lower when the work engagement of a leader is higher than that of a follower rather than when the work engagement of the follower is higher than that of the leader.

#### Conscientiousness as a Moderator of the Incongruence Effect on LMX

High-conscientiousness individuals are intrinsically motivated, enthusiastic about their jobs, and engaged more energy in work (Kim et al., 2009). Literature has suggested that employees who invest more effort and energy in work are more likely to fulfill job tasks assigned by leaders, which then facilitate positive interactions and help the development of LMX (Song et al., 2016). In addition, prior research conceptually stated that conscientious individuals have high levels of goal self-concordance and are more likely to be intrinsically motivated to pursue their goals (Bakker, 2011). Individuals with this motivation are more likely to fit with leaders in the role processes. Thus, conscientiousness may be an important individual disposition in influencing the motivation to fulfill the role demands and the relationship between work engagement incongruence and LMX.

In the present study, we argue that the negative effect of incongruence in leader and follower work engagement on LMX will be mitigated for employees with high levels of conscientiousness. On the one hand, when followers engage more energy, dedication, and absorption in their job tasks than that of their leaders, despite fewer job requirements from supervisors and mutual coordinated interactions, conscientiousness predisposes employees to approach work in ways that can make favorable impressions upon leaders who then tend to treat them with increased respect and trust (Lapierre and Hackett, 2007). Ultimately, the disposition of conscientiousness nurtures social exchanges with leaders and promotes LMX quality. In addition, conscientious followers may invest more focus and effort in fulfilling role requirements, which may facilitate the willingness of leaders to interact with and assign additional tasks to those followers and, in turn, promote the development of LMX (Cropanzano et al., 2017). On the other hand, when work engagement of leaders is higher than that of followers, dissatisfaction of leaders with their less-engaged subordinates may be mitigated by the disposition of conscientiousness of followers because conscientious followers are willing to engage in some positive behaviors, such as helping behaviors toward leaders to maintain their positive impressions and to achieve success (Borman et al., 2001). Previous studies suggested that conscientious employees may regard organizational citizenship behaviors (OCB) as effective ways to satisfy individual needs for achievement and success as these behaviors may lead to more rewards and positive evaluations from leaders (Bukhari, 2009). Those rewards and positive evaluations will increase positive role interactions between followers and leaders. Therefore, it is conceivable that the negative relationships between leader and follower work engagement incongruence and LMX are weakened by the disposition of conscientiousness. All in all, we propose:

##### Hypothesis 4

Conscientiousness will moderate the effect of work engagement incongruence on LMX, such that the negative relationship would be weaker when people are more rather than less conscientious.

## Method

### Data and Sample

The current study was approved by the human research ethics committee of the institute for which the authors work and by the institutional review board of the university. Working adults were recruited from a large private construction company in the southeast of China. This company is very famous for its architectural and structural design. At the first time point, the human resources managers distributed questionnaires to 100 leaders and their corresponding subordinates with a cover letter that briefly explained the purpose of the current survey, the voluntary nature of their participation, and the confidentiality and anonymity of their responses. About 75 leaders and 289 followers agreed to participate in the current study (the response rate was 75%), signed the consent form, and then completed the first survey. At this stage, demographical information and work engagement of leaders and followers, as well as follower conscientiousness, were measured. The human resources managers sent the second survey to those employees 3 months later. At this stage, the perception of LMX quality of followers was assessed. After two waves of data collection, the final sample consists of 72 leaders and 273 followers. All leaders and followers were identified according to their actual hierarchical positions in the company. Among the followers, all of them had a college or bachelor degree or above. About 140 (51.3%) were female, and their average organizational tenure was 6.70 years (*SD* = 5.64). The mean age was 25.91 years (*SD* = 23.76). Among the leaders, 36 (50%) were female. The average organizational tenure was 11.36 (*SD* = 7.33). The mean age was 33.43 years (*SD* = 6.56). Missing data were modeled, using the expectation-maximization algorithm, which assumes that data are missing at random (Little and Rubin, 2002)^1^.

### Measures

For both leaders and followers, demographic information (i.e., age, gender, organizational tenure), and work engagement were measured at Time 1. In addition, the followers reported their conscientiousness at Time 1. Three months later (Time 2), the followers rated their perceived LMX. Given that all surveys were measured in China, the translation/back-translation procedures were used to translate the English-based measures into Chinese (Brislin, 1980). We also compared our translated versions of conscientiousness and work engagement with those already developed Chinese versions (John and Srivastava, 1999; Zhang and Gan, 2005) and found no differences. Responses of conscientiousness and LMX of employees were scored on a seven-point Likert scale, ranging from 1 (strongly disagree) to 7 (strongly agree). The measures of work engagement were rated on a seven-point Likert scale, ranging from 0 (never) to 6 (every day).

#### Conscientiousness

Conscientiousness of followers was measured, using an eight-item scale from John et al. (1991). We compared this scale with the mini-marker set developed by Saucier (1994) and found the item “Is easily distracted” was excluded. Thus, this item was deleted in our measurement^2^. A sample item is “I am someone who does things efficiently.” The Cronbach's α for this scale was 0.76.

#### Work Engagement

We used a nine-item scale developed by Schaufeli et al. (2006) to measure the work engagement of leaders and followers. An example item is “At my work, I feel that I am bursting with energy.” The Cronbach's α for employees was 0.96 and for leaders was 0.94.

#### LMX

LMX of followers was measured through a seven-item scale developed by Liden and Maslyn (1998). The original scale included 11 items. Rofcanin et al. (2017) deleted four items and found that the reliability for the seven items was high (α = 0.91). Given that the current study was a part of a large data investigation for work adults, we used a short version of LMX. A sample item is “My supervisor is the kind of person one would like to have as a friend.” Cronbach's α was 0.91.

#### Control Variables

Existing studies have suggested that LMX quality may be related to similarity in leader and follower demographic characteristics, such as age, gender, and organizational tenure (e.g., Epitropaki and Martin, 1999). Therefore, we controlled for the similarity in leader and follower gender, age, and organizational tenure in our research. In particular, similarities in age and organizational tenure were obtained by calculating absolute difference scores between leader age (or tenure) and follower age (or tenure). In addition, we created a dummy variable for gender similarity. We coded 0 when the gender of a leader and a follower was different and 1 when they had the same gender. The same method has been used by other researchers (e.g., Zhang et al., 2012; Qin et al., 2018).

### Data Analysis

#### Cross-Level Polynomial Regressions

To test the congruence and incongruence effects of leader–follower work engagement on perceptions of employees of LMX, cross-level polynomial regressions and response surface modeling were used (Edwards and Parry, 1993; Jansen and Kristof-Brown, 2005). Polynomial regressions can generate three-dimensional response surfaces, which enables us to test the congruence/incongruence effects on outcomes. In the current research, LMX was regressed on five polynomial terms, that is, work engagement of employees (E), work engagement of leaders (L), work engagement squared of employees (E^2^), work engagement squared of leaders (L^2^), work engagement of employees times work engagement of leaders (E X L). The specific formula was as follows:

(1) $$\begin{array}{r} \text{LMX}={\text{b}}_{0}+b_{1}E+b_{2}L+b_{3}E^{2}+b_{4}\left(E\times L\right)+b_{5}L^{2}+e \end{array}$$

In addition, E and L were centered around the pooled grand mean before calculating the second terms, the purpose of which was to reduce multicollinearity.

We examined the slopes and curvatures along both the congruence line (E = L) and the incongruence line (E = –L) based on polynomial regression procedures. The shape of the surface along the congruence line would be generated by substituting the formula for this line into the polynomial regression equation. Similarly, the shape of the surface along the incongruence line can be derived from substituting the formula for this line into the polynomial regression equation.

To ensure a significant congruence effect, the three second-order polynomial terms (i.e., E^2^, E × L, and L^2^) should be jointly significant, and the curvature along the incongruence line differed significantly from zero (Hypothesis 1). The significance of the slope, along the congruence line, is also examined to determine whether LMX is lower or higher when the joint effect of congruence in leader–follower work engagement is aligned at higher vs. lower levels. A positive (vs. negative) slope means that LMX would be higher (vs. lower) when work engagement of leaders and work engagement of employees are congruent at higher than lower levels (Hypothesis 2).

Finally, additional tests were also conducted to examine whether the surface along the incongruence line was symmetric. The symmetry of the surface along the incongruence line depends on the values of the lateral shift, which is calculated by the formula: (b_2_-b_1_)/2^*^(b_3_-b_4_ + b_5_) (Atwater et al., 1998). A significant positive lateral shift and significant positive curvature of the incongruence line mean that outcomes are higher in the region where E < L along the incongruence line, while a significant positive lateral shift, as well as negative curvature, represents that outcomes are lower in the region where E < L along the incongruence line. Similarly, outcomes are higher in the region where E > L along the incongruence line when a significant negative lateral shift combining with a significant positive curvature is reported, whereas lower levels of outcomes are found in the same region when both significantly negative values of the lateral shift and curvature are reported. In the current study, to support Hypothesis 3, given the negative curvature of the incongruence line, the lateral shift should be positive. Such analysis procedures were widely used in previous studies (Zhang et al., 2012; Liu et al., 2019).

#### Moderation Test

To test the moderating effect of conscientiousness, we followed the procedures outlined by Edwards (1996) and Vogel et al. (2016). In particular, we first needed to control the direct effect of conscientiousness before evaluating the interacting effects, and then the new five interactive terms (i.e., E × C, L × C, E^2^ × C, E × L × C, and L^2^ × C) by multiplying conscientiousness with each of the five polynomial terms. The five newly created terms collectively represent the interactive effects of leader–follower work engagement incongruence and conscientiousness. A significantly incremental explained variance (i.e., a significantly changed *F*-statistic) from the two regression models means a significant interactive effect of conscientiousness. The specific formulas are as follows:

(2) $$\begin{array}{r} \text{LMX}={\text{b}}_{0}+b_{1}E+b_{2}L+b_{3}E^{2}+b_{4}\left(E\times L\right)+b_{5}L^{2}+b_{6}C+e \end{array}$$

(3) $$\begin{array}{r} \text{LMX}={\text{b}}_{0}+b_{1}E+b_{2}L+b_{3}E^{2}+b_{4}\left(E\times L\right)+b_{5}L^{2}+b_{6}C\\ +{\text{b}}_{7}\left(E\times C\right)+b_{8}\left(L\times C\right)+b_{9}\left(E^{2}\times C\right)+b_{10}\left(E\times L\times C\right)+b_{11}\left(L^{2}\times C\right)+e \end{array}$$

The significance of the interactive effects of conscientiousness depends on the incremental explained variance of Equation (3) compared with Equation 2, which is indicated by the significance of changed *F*-statistic. A significant changed *F*-statistic means that conscientiousness would moderate the joint effects of leader–follower incongruence in work engagement on LMX (Edwards, 1996). Because we focus on the moderating effect of conscientiousness in the association between leader–follower work engagement incongruence and LMX, the significance of the slope and curvature along the incongruence line is of importance. When conscientiousness is high, both an insignificant slope and curvature of the surface along the incongruence line would indicate that conscientiousness mitigates the detrimental effect of incongruence in work engagement on LMX.

## Results

### Preliminary Analysis

Means, standard deviations, reliabilities, and intercorrelations among all variables are shown in Table 1. As shown in Table 1, work engagement of both employees and leaders was positively correlated with LMX (*r* = 0.62, *p* < 0.01, and *r* = 0.50, *p* < 0.01, respectively). Conscientiousness of employees was positively correlated with their work engagement (*r* = 0.52, *p* < 0.01), work engagement of leaders (*r* = 0.39, *p* < 0.01), and perception of LMX (*r* = 0.43, *p* < 0.01). To examine the distinctiveness of our hypothesized model, a confirmatory factor analysis was conducted, using Mplus 8.1. Results showed that the four-factor model was acceptable, $\chi_{\left(489\right)}^{2}$ = 1,192.62, *p* < 0.001, CFI = 0.90, TLI = 0.90, RMSEA = 0.07, SRMR = 0.05. We also conducted a series of measurement model comparisons, which are shown in Table 2. These results supported the discriminant validity of our hypothesized model.

**Table 1:** Mean, deviations, and correlations for all variables.

	***M***	***SD***	**1**	**2**	**3**	**4**	**5**	**6**	**7**
1. Gender dissimilarity	0.60	0.49	–						
2. Age dissimilarity	4.97	4.31	0.09	–					
3. Organizational tenure similarity	6.04	5.12	0.05	0.83^**^	–				
4. Employee work engagement	4.57	1.08	0.07	0.40^**^	0.46^**^	(0.96)			
5. Leader work engagement	4.91	0.88	0.04	0.42^**^	0.49^**^	0.64^**^	(0.94)		
6. LMX	5.78	0.91	0.13^*^	0.33^**^	0.38^**^	0.62^**^	0.50^**^	(0.92)	
7. Conscientiousness	3.92	0.49	−0.01	0.17^**^	0.22^**^	0.52^**^	0.39^**^	0.43^**^	(0.77)

*For employees, N = 273. For leaders, N = 72. Reliability coefficients were reported along the diagonal*.

* *p <0.05*.

** *p <0.01*.

**Table 2:** Measurement model comparisons.

**Models**	***χ^2^***	***df***	***χ^2^/df***	**CFI**	**TLI**	**SRMR**	**RMSEA**
1. Hypothesized four-factor model	1,192.62^***^	489	2.44	0.90	0.90	0.05	0.07
2. Three-factor model (leader and follower work engagement were combined)	2,102.30^***^	492	4.27	0.77	0.75	0.08	0.11
3. Three-factor model (follower work engagement and conscientiousness were combined)	1,423.55^***^	492	2.89	0.87	0.86	0.06	0.08
4. Three-factor model (follower work engagement and LMX were combined)	1,916.62^***^	492	3.90	0.80	0.78	0.07	0.10
5. Two-factor model (follower work engagement, conscientiousness, and LMX were combined)	2,134.40^***^	494	4.32	0.76	0.75	0.08	0.11
6. Two-factor model (leader work engagement, follower work engagement and follower conscientiousness were combined)	2,349.92^***^	494	4.76	0.73	0.71	0.09	0.12
7. Single-factor model	3,047.11^***^	495	6.16	0.63	0.61	0.10	0.14

*For employees, N = 273. For leaders, N = 72. CFI, comparative fit index; TLI, Tucker–Lewis index; SRMR, standardized root mean square residual; RMSEA, root mean square error of approximation*.

*** *p <0.001*.

### Hypothesis Testing

**Figure 2:**
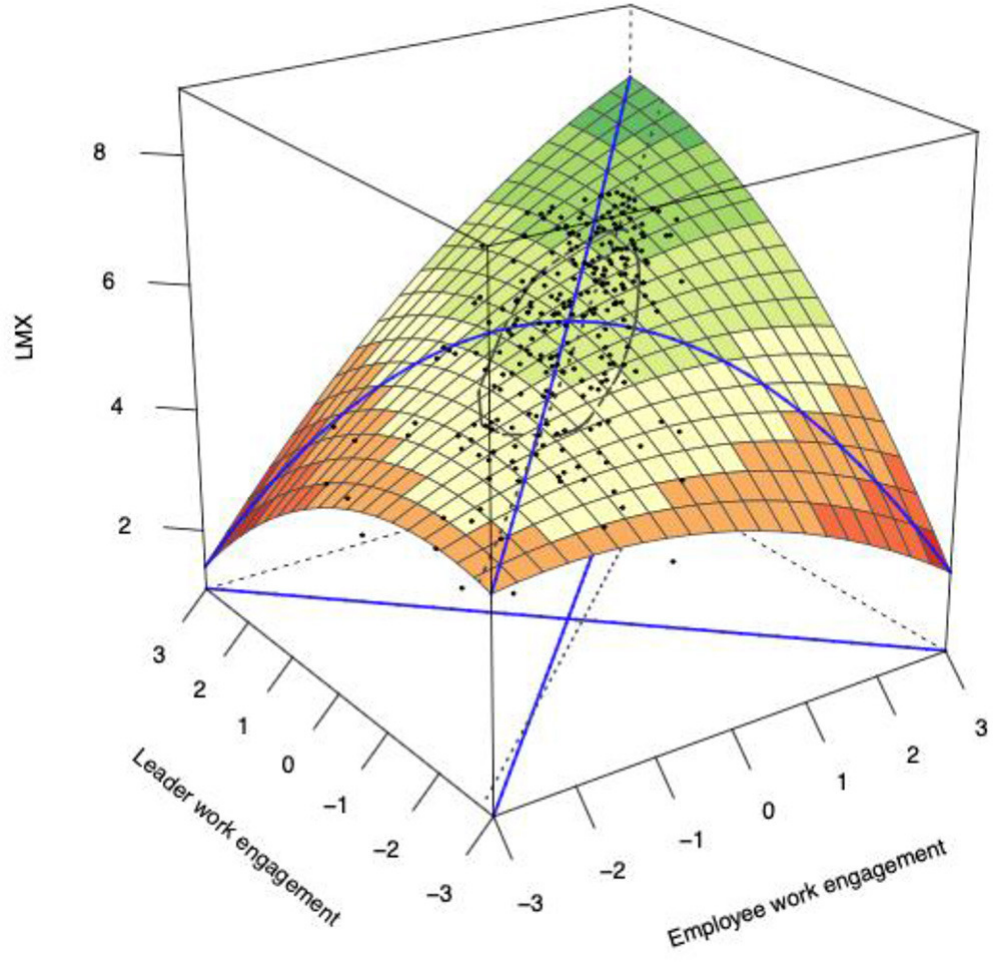
Congruence (incongruence) effects of follower-leader work engagement on LMX.

The results associated with estimated coefficients of main effects (including E, L, E^2^, E × L, and L^2^) and interaction effects (including E × C, L × C, E^2^ × C, E × L × C, and L^2^ × C) for the cross-level polynomial regressions are presented in Table 3. Table 4 shows the values of slopes and curvatures along both the congruence and incongruence lines regarding both main effects and interaction effects (i.e., above and below 1 SD). The response surface is depicted in Figure 2. Hypothesis 1 assumed that LMX quality was higher when leader and follower work engagement was congruent than incongruent. As shown in Tables 3, 4, the three second-order terms were jointly significant, *F* = 3.43, *p* < 0.05. In addition, the curvature, along the incongruence line, was also significant (−0.44, *p* < 0.01). As shown in Figure 2, the surface was downward, indicating that it was an inverted U-shaped one along the incongruence line. The negative curvature and the inverted U-shaped surface, along the incongruence line, indicated that LMX was higher when follower work engagement was aligned with leader work engagement, and any deviation from the congruence line decreased LMX quality. Thus, Hypothesis 1 was supported.

**Table 3:** Cross-level polynomial regression results and path analysis results.

**Variables**	**Main effects**				**Interaction effects**			
	**Model 1**		**Model 2**		**Model 3**		**Model 4**	
	**β**	***SE***	**β**	***SE***	**β**	***SE***	**β**	***SE***
Constant	5.78^**^	0.04	5.83^**^	0.07	5.85^**^	0.07	5.79^**^	0.08
Employee work engagement (E)	0.43^**^	0.05	0.37^**^	0.06	0.32^**^	0.07	0.29^**^	0.07
Leader work engagement (L)	0.18^*^	0.06	0.22^**^	0.07	0.20^**^	0.07	0.27^**^	0.08
Conscientiousness (C)					0.23^*^	0.10	0.06	0.15
E^2^ (b_3_)			−0.09^*^	0.04	−0.09^*^	0.04	−0.06	0.07
E × L (b_4_)			0.23^**^	0.07	0.22^**^	0.07	0.09	0.12
L^2^ (b_5_)			−0.12	0.07	−0.13^*^	0.10	−0.01	0.08
E × C							0.35^**^	0.14
L × C							−0.31^*^	0.17
E^2^ × C							0.22^*^	0.10
E × L × C							−0.45^*^	0.17
L^2^ × C							0.20	0.15
*R* ^2^		0.41		0.42		0.44		0.46
*ΔR* ^2^				0.02				0.03
Change in *F*-statistic				3.43^*^				2.47^*^

* *p <0.05*,

** *p <0.01*.

**Table 4:** The results of slopes and curvatures along the congruence and incongruence lines.

	**Main effects**	**Interaction effects**	
		**−1 SD (*N* = 62)**	**+1 SD (*N* = 64)**
**Congruence (E** **=** **L) line**			
Slope	0.59^**^	0.86^**^	1.02^**^
Curvature	0.03	0.12	0.02
**Incongruence (E** **=** **–L) line**			
Slope	0.15	−0.16	0.34
Curvature	−0.44^**^	−0.61^**^	0.24

** *p <0.01*.

Hypothesis 2 assumed LMX quality was higher when leader and follower work engagement was aligned at higher rather than lower levels. In supporting this hypothesis, a positively significant slope along the congruence line was reported (0.59, *p* < 0.01). To test Hypothesis 3, we first calculated the lateral shift. In this study, the lateral shift value was 0.18, indicating that LMX was lower in the region where work engagement of followers was lower than the work engagement of leaders. It means that LMX quality is lower when the work engagement of a leader is higher than that of a follower. Thus, Hypothesis 3 was supported.

### Moderation Test

To test the moderating effects of conscientiousness in the relationship between leader and follower work engagement incongruence and LMX, we first tested the significance of the five third-order terms (i.e., E × C, L × C, E^2^ × C, E × L × C, and L^2^ × C). As shown in Table 3, a significant change in *F*-statistic was reported, *F* = 2.47, *p* < 0.05, indicating a significant interactive term of conscientiousness and work engagement incongruence in predicting LMX. Given that we focus on the moderating effect of conscientiousness in the association between leader–follower work engagement incongruence and LMX, the values of the slope and the curvature along the incongruence line are of importance. Then, we tested how the slopes and curvatures of the surface along the incongruence line varied across different levels of conscientiousness. The variable of conscientiousness was divided into high and low levels, using a Mean split. The data above +1 SD were categorized as “high” levels, and below −1 SD were categorized as “low” levels. As shown in Table 4, when employees were less conscientious, the curvature of the surface was significant (curvature = −0.61, *p* < 0.01), indicating that the relationship between leader and follower work engagement incongruence and LMX was negative. In contrast, when employees were more conscientious, the negative effect of work engagement incongruence was mitigated. Neither a significant value of a slope nor a significant value of curvature was reported (slope = 0.34, *p* > 0.05 curvature = 0.24, *p* > 0.05), supporting Hypothesis 4 that conscientious people were less likely to perceive low LMX quality when facing a misfit in leader–follower work engagement.

## Discussion

Despite research on work engagement, most studies focused on how work engagement was in relation to in-role or extra-role performance, neglecting the relationship between work engagement, and LMX. To address this research gap, we draw upon person-environment fit theory and LMX theory to test whether (in)congruence in leader and follower work engagement would influence perceptions of followers of LMX quality, and whether the disposition of the followers of conscientiousness would weaken the negative relationship between leader and follower work engagement incongruence and LMX. By using self-reported measures of leaders and followers with a two-wave survey, the results showed that, compared with incongruence in leader and follower work engagement, congruence in leader and follower work engagement resulted in higher levels of LMX. In addition, perceptions of the followers of LMX quality varied across different levels of work engagement congruence and incongruence. Specifically, regarding the congruence, LMX was higher when leader and follower work engagements were aligned at higher rather than lower levels; regarding the incongruence, LMX was higher when follower work engagement was higher than leader work engagement than when follower work engagement was lower than that of a leader. Moreover, dispositions of the followers of conscientiousness moderated the effect of incongruence in leader and follower work engagement on LMX. Conscientious followers may perceive higher levels of LMX even though their work engagement was misaligned with their leaders.

Theoretical implications. The present research has several theoretical implications. First, researchers have indicated that work engagement is beneficial for job satisfaction, job performance, and well-being (Breevaart et al., 2015; Bakker and Albrecht, 2018). The current study, enriching the effects of work engagement, indicates that work engagement would influence social relationships with leaders (i.e., LMX). This is one of the first studies that establish the relationship between work engagement and social relationships with important others (e.g., leaders) at work. Drawing from person-environment fit theory and LMX theory, our results suggested that leader and follower work engagement would jointly influence perceptions of followers of LMX quality. Specifically, congruence in leader and follower work engagement would be beneficial for the development of LMX, while incongruent work engagement would result in low levels of LMX.

Second, our findings challenge the prevailing consensus on the universal benefits of work engagement across all individuals and contexts by taking both leader and follower work engagement into consideration. Our results showed that low work engagement levels of followers were not necessarily detrimental to fostering positive social relationships with leaders. When leader and follower work engagements are congruent at low levels, the similarity will increase coordinated interactions between leaders and followers (Metiu and Rothbard, 2013; Costa et al., 2014). Those positive interactions will further enhance LMX by promoting mutual trust and attraction (Chen et al., 2016). In contrast, high levels of work engagement of followers are not always good for promoting LMX, which varies depending on the levels of work engagement of leaders. When work engagement of followers is high while work engagement of their leaders is low, the followers are less likely to receive supervisor support and engage in positive interactions with the leaders (Marstand et al., 2017b), resulting in a weak sense of LMX. This study provided a new perspective to help us understand the effects of work engagement on LMX. That is, both leader and follower work engagements should be considered as joint factors in influencing LMX.

Third, this study contributes to the work engagement and conscientiousness literature (Marstand et al., 2017a) by revealing that conscientiousness would moderate the relationship between leader and follower incongruence in work engagement and LMX. Specifically, the results show that work engagement incongruence would not result in low levels of LMX when followers have high levels of conscientiousness. A considerable amount of research has investigated conscientiousness as either an independent variable in predicting various work-related outcomes, such as job performance and LMX (Lapierre and Hackett, 2007; Chae et al., 2018) or a moderator in strengthening or weakening other relationships (Mawritz et al., 2014), overlooking its roles in the effects of a person-environment misfit on outcomes. Our study, by considering the nature of conscientiousness and the person-supervisor misfit in work engagement, documents that conscientiousness is an important buffer against the detrimental effect of incongruence in work engagement on LMX.

## Practical Implications

Our findings also provide several important managerial implications. First, to maintain or/and improve close relationships with leaders, it is crucial for the followers to be aware of the extent of their work engagement of leaders and keep aligned with the work engagement of their leaders at the same level (Gutermann et al., 2017). Congruence in leader and follower work engagement would bring a number of benefits to followers. Our results showed that this congruence would increase the perceptions of followers of LMX. LMX has been suggested to relate to various positive outcomes, such as increased OCBs and job performance (Dulebohn et al., 2012). In addition, this congruence and similarity between a leader and a follower may motivate leaders to provide more resources, such as trust, rewards, and autonomy, for followers (Bauer and Green, 1996), which will help employees achieve career success and self-worth at work. As such, the employees should be highly alert to the extent to which their leaders engage themselves in work. When being aware that their leaders put much effort, time, and energy into work tasks, followers should keep aligned with their leaders. Although a low level of work engagement congruence is beneficial for the development of LMX, we do not recommend employees to engage less energy, dedication, and absorption in their work roles because low levels of work engagement will result in various negative outcomes, such as lower job performance and OCB (Breevaart et al., 2015; Harju et al., 2016). In addition, LMX quality is lower when a leader and a follower are congruent at lower than higher levels of work engagement, indicating that high rather than low levels of work engagement are the best for employees to develop LMX. Thus, to achieve the best quality of LMX and other positive outcomes, employees should increase their work engagement and keep align with their leaders at high levels.

Second, given that conscientiousness can moderate the relationship between incongruence in work engagement and LMX, followers should try to enhance their levels of conscientiousness. Some research has characterized personal traits, such as conscientiousness as stable over time (McCrae and Costa, 2008). Other researchers, however, state that personal traits can shift through proper training programs (Magidson et al., 2014). A bottom-up approach that requires employees to schedule their activities into specific periods is effective to enhance conscientiousness of one because this approach increases individual engagement in goal-directed activities (Magidson et al., 2014). In addition, managers and organizations should provide employees with greater autonomy in decision-making, which may enable employees to feel responsible and conscientious to work demands (Van Yperen et al., 2016).

## Limitations and Future Research

In spite of its theoretical and practical implications, this study has several limitations. First, given our research design, the problem associated with inferring causality is a major limitation of our study. Indeed, LMX may act as the antecedent of leader and follower work engagement alignment. High levels of LMX mean that a leader and a follower would have positive interactions (Pan and Lin, 2018). In this condition, the follower is more likely to learn how the leader engages his/her energy, absorption, and dedication in work tasks, and keeps aligned with the leader (Bandura, 1977; Gutermann et al., 2017). Thus, congruence in leader and follower work engagement may occur. Therefore, to determine the causality between (in)congruence in leader and follower work engagement and LMX, future studies can test such an effect, using longitudinal and/or experimental research design rather than time-lagged research design (Bakker, 2014).

Second, the data for this study were collected from the employees working in a Chinese firm. Given that Chinese culture is characterized by a high degree of power distance (Carl et al., 2004), leaders may have a greater influence on the behaviors of the followers in such a culture. Thus, the followers are more likely to keep consistent with their leaders in terms of work state such as work engagement. We recommend future research could address this limitation by comparing findings based on samples from different power distance cultures.

Third, although we measured variables from different sources and a time-lagged research design, self-reported data may still suffer from common method biases. Future studies can verify and strengthen the findings of the current study by using both leader- and follower-rated LMX. In addition, future research should explore whether congruence and incongruence in leader and follower work engagement influence the perceptions or behaviors of leaders. When leader and follower work engagements are aligned at the same levels, the leader may evaluate his or her followers as attractive and allocate more valuable resources to them (Thompson et al., 2006). Moreover, the incongruence in leader and follower work engagement does not necessarily impair the assessment of followers of leaders, especially when work engagement of the leaders is lower than that of their followers because those leaders may be willing to work with their engaged followers to fulfill more responsibilities that leaders should have.

Fourth, our conclusions of the congruence and incongruence effects may be inflated because of the small sample size. Although many researchers conducted cross-level polynomial regression, using a small sample size (Jansen and Kristof-Brown, 2005; Cole et al., 2013), a larger sample should be better when conducting response surface analysis because many higher-level equations are estimated. Thus, further research could replicate current research, using a larger sample.

## Conclusion

Although researchers explored the various work-related benefits of work engagement (Bakker and Albrecht, 2018), the effect of work engagement on LMX has not been examined. The present study investigated the effects of leader and follower (in)congruence in work engagement on LMX and the moderating effect of conscientiousness in the relationship between leader and follower incongruence in work engagement and LMX. By using cross-level polynomial regressions and response surface modeling, the results showed that LMX was higher when leader and follower work engagements were congruent rather than incongruent. In addition, LMX quality was perceived differently across different levels of congruence/incongruence in leader and follower work engagement. Regarding the congruence, followers whose work engagement was highly aligned with that of their leaders would be more likely to experience higher LMX quality than the congruence at lower levels. Regarding the incongruence, when the followers engaged less vigor, dedication, and absorption in their work than their leaders, they were more likely to experience lower levels of LMX. We also found that the effect of incongruence in work engagement on LMX was contingent on the conscientiousness of the followers. In particular, conscientiousness would mitigate the adverse effects of incongruence in leader and follower work engagement on LMX.

## Data Availability Statement

The raw data supporting the conclusions of this article will be made available by the authors, without undue reservation.

## Ethics Statement

The studies involving human participants were reviewed and approved by the Human Research Ethics Committee of the institute. All procedures performed in studies involving human participants were in accordance with the APA ethical principles of psychologists and code of conduct. The patients/participants provided their written informed consent to participate in this study.

## Author Contributions

YY: conceptualization, investigation, writing of the original draft, editing, analysis, and funding acquisition. ZW: revising the manuscript and providing thoughtful suggestions about the manuscript. XL: data curation, resources, and editing.

## Conflict of Interest

The authors declare that the research was conducted in the absence of any commercial or financial relationships that could be construed as a potential conflict of interest.

## Publisher's Note

All claims expressed in this article are solely those of the authors and do not necessarily represent those of their affiliated organizations, or those of the publisher, the editors and the reviewers. Any product that may be evaluated in this article, or claim that may be made by its manufacturer, is not guaranteed or endorsed by the publisher.

## Footnotes

^1^Additional sensitivity analyses by using listwise deletion were also conducted. The virtually same results were obtained as those reported in the current manuscript.

^2^To ensure the construct validity of conscientiousness, we recruited additional 152 work adults to complete the measures of the scale from John et al. (1991) and the scale we used in this study. A confirmatory factor analysis was conducted, using Mplus 8.1. The results showed that the one-factor model, loading 17 items on one latent factor [$\chi_{\left(119\right)}^{2}$ = 422.48, *p* < 0.001, CFI = 0.69, TLI = 0.64, RMSEA = 0.13, *p* < 0.001], were not significantly different from the two-factor model, loading items from John et al. (1991) and the scale used in this study on two different factors [$\chi_{\left(118\right)}^{2}$ = 422.06, CFI = 0.69, TFI = 0.64, RMSEA = 0.13, *p* < 0.001], Δχ2 [Δ*df* = 1] = 0.02, *p* > 0.05. Thus, we could conclude that the scale used in this study was not different from the scale from John et al. (1991). In addition, a high correlation between the scale we used and the nine-item scale (*r* = 0.81, *p* < 0.01) was reported. These results validated the scale for measuring conscientiousness is appropriate in this study.
